# A Sea Mud Feed Matrix Shapes Short-Term Dietborne Glyphosate Exposure in the Sea Cucumber (*Apostichopus japonicus*): Tissue Residues, Buffered Enzyme Responses, and Dominance-Structured Gut Microbiota Shifts

**DOI:** 10.3390/ani16091344

**Published:** 2026-04-28

**Authors:** Jingchun Sun, Libin Zhang, Christopher D. Hepburn, Shaoping Kuang, Hongsheng Yang

**Affiliations:** 1 College of Environment and Safety Engineering, Qingdao University of Science and Technology, Qingdao 266042, China; 2 Laboratory of Marine Ecology and Environmental Sciences, Institute of Oceanology, Chinese Academy of Sciences, Qingdao 266000, China; 3 Laboratory for Marine Ecology and Environmental Science, Qingdao Marine Science and Technology Center, Qingdao 266237, China; 4 State Key Laboratory of Breeding Biotechnology and Sustainable Aquaculture, Institute of Oceanology, Chinese Academy of Sciences, Qingdao 266000, China; 5 Department of Marine Science, University of Otago, Dunedin 9054, New Zealand

**Keywords:** glyphosate, *Apostichopus japonicus*, dietborne exposure, sea mud feed matrix, enzyme biomarkers, gut microbiota

## Abstract

Deposit-feeding sea cucumbers ingest sediment-like particles, making substrate-associated exposure pathways ecologically relevant in coastal aquaculture. In this study, a sea mud feed matrix was used to evaluate short-term dietborne/substrate-linked glyphosate exposure in *Apostichopus japonicus* over 72 h, with the aim of characterizing early residue formation, short-term sublethal biomarker responses, and gut microbiota shifts under a benthic feeding scenario. Analytical verification confirmed a clear glyphosate gradient in the prepared feed matrices, with no glyphosate detected in the control matrix and measured concentrations of 8.66 ± 1.59 mg/kg, 1330 ± 390 mg/kg, and 6960 ± 1710 mg/kg in the low-, medium-, and high-dose groups, respectively. No mortality or obvious external lesions were observed during the exposure period. Tissue analysis confirmed measurable internal glyphosate residues and compartment-specific distribution, indicating successful internal exposure under the matrix-linked route. Most digestive and immune/antioxidant biomarkers remained relatively stable within the 72 h window; however, amylase showed a marked response in the low-dose group, and superoxide dismutase showed dose-associated changes in the medium- and high-dose groups, indicating selective sensitivity among enzyme endpoints. Gut microbiota analysis revealed a dominance-structured community with limited alpha-diversity variation among groups, whereas community composition showed subtle treatment-related shifts that were more evident at finer taxonomic resolution. Predicted functional profiles remained broadly similar across treatments. Overall, the 72 h exposure design was effective for identifying early internal exposure and short-term biological responses under a sea mud-associated feeding route, while host physiological responses remained largely buffered over this time scale and the gut microbiota provided a more sensitive interface-level signal of exposure-associated change. These findings support the value of a route-specific, gut-centered framework for evaluating early herbicide exposure responses in benthic mariculture species and suggest that matrix-associated feeding conditions may modify the apparent magnitude of short-term responses.

## Simple Summary

Sea cucumbers are deposit feeders that naturally ingest sediment-like particles, so substrate-linked contaminant exposure is relevant to benthic aquaculture. In this study, we used a sea mud feed matrix to examine short-term glyphosate exposure in the sea cucumber *Apostichopus japonicus* over 72 h. This design was intended to capture early internal exposure and short-term biological responses under a sediment-associated feeding scenario rather than long-term toxicity. The prepared feed matrices showed a clear glyphosate gradient, and tissue analysis confirmed measurable internal residues after exposure. No mortality or obvious external lesions were observed during the experiment. Most digestive and immune/antioxidant enzyme responses remained relatively stable within this short time window, although amylase and superoxide dismutase showed treatment-related changes. Gut microbiota analysis indicated limited changes in overall diversity but detectable shifts in community structure. These results show that a sea mud-associated feeding route can produce measurable internal glyphosate exposure while yielding relatively buffered short-term host responses, with the gut microbiota providing a more sensitive signal of exposure-associated change.

## 1. Introduction

Deposit-feeding sea cucumbers continuously ingest sediment-like particles, making sediment-associated exposure pathways ecologically realistic for this benthic group [1,2]. Among farmed holothurians, *Apostichopus japonicus* is a representative species whose physiological condition and stress responsiveness are closely associated with intestinal function and the gut-associated microbiota [3,4,5]. Because deposit feeding couples particle ingestion with gut transit, the intestine functions not only as a digestive organ but also as a dynamic interface where particulate-bound chemicals and sediment-derived microbes interact with host epithelial barriers and innate immune processes [4,5,6,7]. Accordingly, digestive and immune/antioxidant enzyme activities, together with 16S rRNA-based gut microbiota profiling, are increasingly used to evaluate sublethal stress and health status in sea cucumbers under aquaculture-relevant challenges [6,7,8,9].

Herbicide contamination has emerged as a relevant concern for coastal mariculture systems influenced by watershed inputs. Extensive use of glyphosate (N-(phosphonomethyl)glycine) and the widespread occurrence of its main metabolite aminomethylphosphonic acid (AMPA) across soils, surface waters, and sediments have been documented in environmental reviews and monitoring studies, highlighting realistic transport routes from agricultural and urban sources to nearshore environments [10,11,12]. In coastal zones, episodic pulses driven by rainfall/runoff and hydrodynamic redistribution can deliver polar contaminants to both the water column and bottom sediments, creating mixed exposure scenarios for benthic invertebrates [11,12,13]. Importantly, glyphosate’s high polarity and strong interactions with particles and metals can complicate extraction and quantification, especially in high-salinity or particle-rich matrices; continued analytical improvements have therefore been important for reliable exposure confirmation of glyphosate/AMPA [14,15,16]. These lines of evidence motivate mechanistic assessments under exposure routes that reflect how benthic farmed animals encounter contaminants in practice, rather than relying solely on idealized waterborne paradigms.

Glyphosate’s canonical mode of action is inhibition of 5-enolpyruvylshikimate-3-phosphate synthase (EPSPS) in the shikimate pathway, which is absent in metazoans but present in plants, fungi, and many bacteria [17,18]. While this provides a microbiome-relevant background for indirect host effects [17,18,19,20], the magnitude and detectability of host responses are expected to depend strongly on exposure form, particularly when glyphosate partitions to particles and sediments. Glyphosate exposure has been linked to microbial community perturbation in diverse animals, and microbiome-associated host effects have been increasingly discussed in both experimental and environmental contexts [20,21,22]. In aquatic organisms, sublethal glyphosate exposure has been associated with oxidative, immune-related, neurochemical, and metabolic disturbances, supporting the use of biochemical biomarkers for early-response assessment [23,24,25]. Evidence is also accumulating that chronic or repeated exposure can reshape host-associated microbiota in fish, supporting the sensitivity of microbiome endpoints for herbicide stress [26].

In sea cucumbers, non-specific immune parameters, antioxidant defenses, and digestive enzyme activities are widely recognized as responsive to environmental challenges, and these biomarkers are routinely used in health assessment and stress physiology studies [6,7,8,9,27]. Intestinal microbiota has likewise been implicated in development, feeding transitions, and physiological plasticity, including early-life core microbiome establishment and deterministic assembly processes during gut perturbation/regeneration [3,5,28]. These studies collectively support a gut-centered view of holothurian stress biology, in which changes in digestive capacity, redox balance, and innate immune effector activity can co-occur with microbial restructuring. However, the exposure route is likely to shape effect magnitude and detectability, and this issue is especially relevant when comparing waterborne exposure with dietary/substrate-linked exposure in deposit-feeding species.

Why might substrate-linked (dietary/sediment-associated) glyphosate exposure produce more attenuated or less overt host responses than waterborne exposure, even when internal residues are detectable? First, bioaccessibility may be constrained by matrix binding: glyphosate adsorbs to mineral surfaces and metal (hydr)oxides and competes with phosphate for adsorption sites, which may reduce the freely available fraction and slow uptake from particulate-associated sources [29,30,31]. Second, deposit feeders ingest particles that pass through a buffered gut environment; mucus, variable pH, and digestive secretions may further modulate desorption/solubilization, potentially spreading exposure over time and dampening acute biomarker excursions relative to direct waterborne contact across epithelia [6,7,29]. Third, microbial endpoints can be misleading if interpreted with naïve diversity metrics alone. 16S datasets are compositional and can present “low-diversity” patterns when one or a few taxa dominate; biologically meaningful changes may occur at the level of dominance structure, taxon replacement, or log-ratio balances even when alpha-diversity indices change little [32,33,34]. This is relevant for deposit feeders, where strong dominance by specific bacterial lineages can be common and may mask exposure-associated community restructuring if analyses do not account for compositional and dominance-aware principles [28,33,34]. Finally, modest physiological shifts may still be aquaculturally meaningful: attenuated enzyme responses do not imply “no effect,” but may indicate a low-bioaccessibility exposure regime in which host adjustment and microbiota re-organization occur without the pronounced oxidative/immune bursts typically observed under waterborne challenge models [23,24,25,26,27,30,31].

Against this backdrop, the present study focuses on dietary/substrate-linked glyphosate exposure in *A. japonicus* using a sea mud feed matrix to better approximate sediment- and bottom-material-associated exposure conditions in sea cucumber farming. We integrate exposure confirmation (glyphosate/AMPA residues) with digestive enzyme activities, immune/antioxidant enzyme responses, and 16S rRNA-based gut microbiota profiling to build a coherent, gut-centered interpretation of physiological adjustment under a low-bioaccessibility exposure route. We specifically test the following hypotheses/research questions: (i) dietary/substrate-linked glyphosate exposure yields measurable internal residues but elicits attenuated enzyme responses relative to typical waterborne paradigms because matrix binding constrains bioaccessibility and slows uptake [11,12,13,29,30,31]; (ii) even when alpha-diversity changes are small (or uniformly low due to dominance), dietary exposure still drives interpretable shifts in gut microbiota dominance structure and taxon composition when analyzed with compositional principles [28,32,33,34]; and (iii) variation in digestive versus immune/antioxidant enzyme activities co-varies with microbiota dominance patterns, supporting gut-centered coupling between microbial restructuring and host physiological adjustment under low-bioaccessibility exposure [6,7,8,9,26,27,28]. By structuring this study around route-specific hypotheses, we aimed to determine whether a 72 h sea mud-associated dietary exposure could reveal early internal glyphosate residues, short-term sublethal biomarker responses, and gut microbiota shifts in *A. japonicus*, thereby providing exposure route-relevant evidence for herbicide risk evaluation in a benthic mariculture species.

## 2. Materials and Methods

### 2.1. Animals, Acclimation and Experimental Layout

Healthy Japanese sea cucumbers (*Apostichopus japonicus*) were obtained from a commercial farm in Rushan, Shandong, China. The experimental animals were two-year-old sea cucumbers with a body mass of 80–100 g. They were transported to the laboratory and acclimated for 3 days in independent 65 L plastic tanks (buckets). During acclimation, sea cucumbers were not fed. The experiment was conducted using filtered natural seawater under laboratory conditions. Seawater temperature was maintained at 12–14 °C, continuous aeration was provided, salinity and dissolved oxygen were maintained within the normal range for *A. japonicus* culture, and seawater was renewed every 24 h.

After acclimation, animals were randomly assigned to four treatment groups: a control diet group (C) and three glyphosate-spiked sea mud feed groups representing low-, medium-, and high-dose treatments (L, M, and H). Each group consisted of four independent replicate tanks, with 10 sea cucumbers per tank (40 individuals per treatment in total), and the exposure duration was 72 h. At the 72 h endpoint, individuals were sampled without pooling, and all samples represented individual sea cucumbers. For each treatment group, a total of 10 individuals were randomly collected and numbered 1–10, with sampling performed across all replicate tanks (i.e., each tank contributed sampled individuals). All downstream assays were conducted on randomly selected subsamples drawn from this collected set. Specifically, glyphosate residue quantification was performed with *n* = 3 per group, enzyme activity assays were performed with *n* = 5 per group, and 16S rRNA gene sequencing was performed with *n* = 5 per group. Throughout the experiment, tanks were continuously aerated, husbandry conditions were kept consistent across treatments, and a 12 h light:12 h dark photoperiod was maintained. No mortality or obvious skin lesions were observed over the 72 h exposure period.

### 2.2. Preparation of Sea Mud Feed Matrix and Glyphosate Dosing Rationale

To mimic a benthic, sediment-associated ingestion route that is ecologically relevant for deposit-feeding holothurians, a sea mud-based feed matrix was used as a sediment-like carrier for dietary exposure. Briefly, sea mud and commercial formulated feed were combined at a mass ratio of 3:1 (sea mud:feed) and thoroughly homogenized with seawater to generate a cohesive matrix suitable for ingestion by sea cucumbers. This design intentionally introduced a particulate phase to distinguish the dietary/substrate-linked exposure scenario from a purely waterborne exposure design.

Glyphosate reagent powder (catalog no. N817057-100g; Macklin Biochemical Co., Ltd., Shanghai, China; purity 95%) was incorporated into the sea mud feed matrix to generate the treatment diets. The control (C), low-dose (L), medium-dose (M), and high-dose (H) groups were prepared with nominal glyphosate addition proportions of 0%, 0.4%, 2%, and 10%, respectively. Because the animals were maintained in group culture buckets and feed intake varied among individuals, the exposure was defined at the treatment diet level rather than as an exact administered dose per individual. Because a sediment-like matrix may adsorb glyphosate and modify its freely available fraction, the sea mud matrix was treated as an active exposure component rather than an inert carrier in the present design. Therefore, the actual glyphosate concentrations in the prepared matrices were verified analytically (Section 2.3.1), and these measured values were used as the operational exposure descriptors for downstream interpretation rather than a simple water-equivalent dose.

### 2.3. Chemical Analytics

#### 2.3.1. Glyphosate in Feed

No glyphosate was detected in the control matrix. The measured glyphosate concentrations in the L, M, and H feed matrices were 8.66 ± 1.59 mg/kg, 1330 ± 390 mg/kg, and 6960 ± 1710 mg/kg, respectively (mean ± SD). These measured concentrations were used as the operational exposure descriptors for downstream interpretation of dietary/substrate-linked glyphosate exposure.

#### 2.3.2. Glyphosate in Tissues

For glyphosate residue analysis, body wall, respiratory tree, intestine, and coelomic fluid samples were collected from randomly selected individuals in each treatment group. Each sample represented an individual sea cucumber, and no pooling was performed. All tissue and body fluid samples were processed immediately after collection or stored at −80 °C until subsequent analysis. Glyphosate residue determination was performed with *n* = 3 biological replicates per treatment group. Subsequent extraction and quantification were carried out according to the analytical procedure described below.

### 2.4. 16S rDNA Sequencing and Bioinformatics

Intestinal contents were collected aseptically from individually sampled sea cucumbers for gut microbiota analysis (*n* = 5 per treatment group). The samples were immediately frozen and stored at −80 °C until DNA extraction. Total genomic DNA was extracted using the HiPure Stool DNA Kit (Magen Biotech Co., Ltd., Guangzhou, China) according to the manufacturer’s instructions.

After DNA extraction, the V3–V4 region of the bacterial 16S rRNA gene was amplified using barcode-tagged primers 341F (5′-CCTACGGGNGGCWGCAG-3′) and 806R (5′-GGACTACHVGGGTATCTAAT-3′), generating an amplicon of approximately 466 bp. PCR products were purified using AMPure XP Beads (Beckman Coulter, Brea, CA, USA) and quantified using a Qubit 3.0 fluorometer (Thermo Fisher Scientific, Waltham, MA, USA). Sequencing libraries were prepared using the Illumina DNA Prep Kit (Illumina, San Diego, CA, USA), and library quality was checked using the ABI StepOnePlus Real-Time PCR System (Applied Biosystems, Foster City, CA, USA). Libraries were then pooled and sequenced on the Illumina NovaSeq 6000 platform in PE250 mode.

Raw sequencing data were subjected to quality filtering, paired-end read merging, tag filtering, OTU clustering, and chimera removal. Specifically, low-quality reads were filtered using FASTP, paired-end reads were merged using FLASH, OTUs were clustered using the UPARSE algorithm in USEARCH, and chimeric sequences were removed using the UCHIME algorithm. The resulting high-quality sequences were defined as effective tags and used for downstream community analysis. After quality control, effective tag counts were on the order of 10^5^ per sample, with effective ratios generally above 90%, and the merged tag length was approximately 466 bp.

Taxonomic annotation and diversity analysis were performed based on the resulting OTU abundance table. Alpha-diversity and beta-diversity analyses were conducted to evaluate group-level differences in the gut microbial structure. LEfSe analysis was used to identify taxa with differential abundance among groups and to visualize their effect size and taxonomic distribution. PICRUSt2 was used to predict potential functional profiles of the intestinal microbiota based on KEGG pathway annotation. Because these functional predictions were inference-based, they were interpreted cautiously.

### 2.5. Enzyme Assays

Digestive enzymes were measured in intestinal tissue collected from individually sampled sea cucumbers (*n* = 5 per treatment group) as functional readouts of digestive capacity under dietary/substrate-linked exposure. Intestinal tissues (approximately 0.1 g) were weighed, homogenized in pre-cooled extraction buffer (1× PBS; Nanjing Jiancheng Bioengineering Institute, Nanjing, China) under cold conditions, and centrifuged at 3500 rpm for 15 min at 4 °C. The supernatants were used for enzyme assays following the kit protocols. The following digestive enzymes were quantified using commercial colorimetric kits (Nanjing Jiancheng Bioengineering Institute, Nanjing, China): alkaline phosphatase (ALP), acid phosphatase (ACP), cellulase (CL), trypsin, lipase (LPS), and amylase (AMS). Activities were normalized according to the kit manuals (e.g., per mg protein where required).

Immune and oxidative stress-related biomarkers were determined in coelomic fluid collected from individually sampled sea cucumbers (*n* = 5 per treatment group) to evaluate systemic functional status under the dietary/substrate-linked exposure route. Coelomic fluid samples were mixed with pre-cooled extraction buffer, processed under cold conditions, and clarified by centrifugation at 3500 rpm for 10 min at 4 °C. The following biomarkers were quantified using commercial kits (Nanjing Jiancheng Bioengineering Institute, Nanjing, China) according to the manufacturer’s instructions: superoxide dismutase (SOD), catalase (CAT), glutathione peroxidase (GSH-PX), glutathione reductase (GR), peroxidase (POD), malondialdehyde (MDA), and lysozyme (LZM). Units and normalization followed the kit manuals and were reported consistently across groups.

### 2.6. Statistical Analysis

Statistical analyses were performed using GraphPad Prism version 8.0 (GraphPad Software, San Diego, CA, USA). A two-sided *p* < 0.05 was used as the primary threshold for statistical significance. When *p* < 0.01 was additionally indicated, it was used only to denote comparatively stronger statistical evidence for a given comparison rather than as a separate significance criterion applied to a different subset of data.

For glyphosate residue data, two-way analysis of variance (ANOVA) was used to evaluate the effects of treatment group and tissue type, as well as their interaction. When appropriate, post hoc multiple-comparison tests were applied to further examine group differences. For digestive enzyme activities and immune/oxidative stress-related biomarkers, one-way ANOVA was used to test for differences among treatment groups. When the assumptions of ANOVA were met, post hoc pairwise comparisons were conducted accordingly.

For gut microbiota analysis, alpha-diversity indices were compared among groups using the Kruskal–Wallis test. Beta-diversity was assessed using Bray–Curtis distance-based principal coordinates analysis (PCoA), and treatment effects on community composition were evaluated using permutational multivariate analysis of variance (PERMANOVA). LEfSe was used to identify taxa with differential abundance among groups, and PICRUSt2 was used for functional prediction. Statistical results were interpreted with emphasis on overall effect patterns and biological relevance while avoiding overreliance on dichotomous interpretation based solely on significance thresholds.

## 3. Results

### 3.1. Clinical Status: Survival and External Phenotype

**Figure 1:**
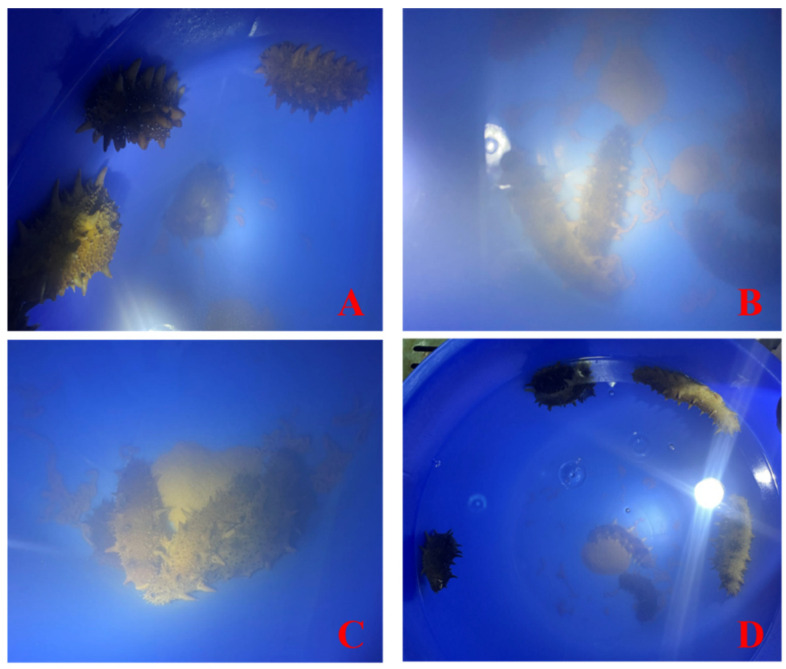
Culture status of sea cucumbers after 72 h exposure. Representative photographs showing the overall culture status of sea cucumbers in C, L, M, and H groups after 72 h dietary/substrate-linked exposure using a sea mud feed matrix. No mortality was observed. Subfigures are described separately: (**A**) C group; (**B**) L group; (**C**) M group; (**D**) H group.

**Figure 2:**
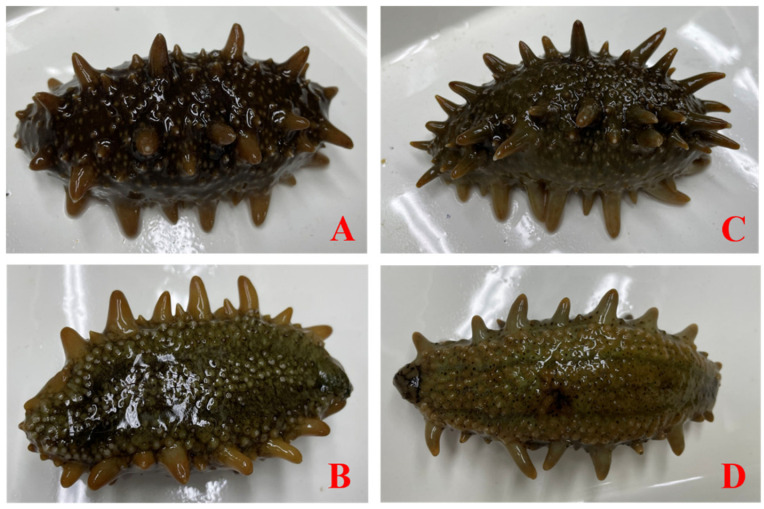
External phenotype of sea cucumbers after 72 h exposure. Representative images of the body wall and ventral surface of sea cucumbers in C and H groups after 72 h exposure. No obvious skin peeling or visible lesions were observed. Subfigures are described separately: (**A**) Dorsal view of a sea cucumber in C group; (**B**) Ventral view of a sea cucumber in C group; (**C**) Dorsal view of a sea cucumber in H group; (**D**) Ventral view of a sea cucumber in H group.

Four experimental groups were established: a control group (C) and three dietary/substrate-linked glyphosate treatment groups representing low-, medium-, and high-dose exposure levels (L, M, and H). Each group consisted of four replicate tanks, with 10 sea cucumbers per tank (40 individuals per treatment in total). Over the 72 h exposure period, no mortality occurred, and no obvious skin peeling or visible lesions were observed in any treatment group (Figure 1 and Figure 2), indicating no overt clinical impairment within this short-term exposure window. At the endpoint, individuals were randomly collected across replicate tanks for downstream measurements, and no pooling was performed. Glyphosate residues were quantified in *n* = 3 individuals per group, whereas enzyme assays and 16S rRNA gene sequencing were conducted in *n* = 5 individuals per group.

### 3.2. Glyphosate Residues

#### 3.2.1. Glyphosate Burden in the Sea Mud Feed Matrix

Analytical verification confirmed a clear treatment gradient in the prepared feed matrices. No glyphosate was detected in the control matrix, whereas the measured concentrations in the L, M, and H feed matrices were 8.66 ± 1.59 mg/kg, 1330 ± 390 mg/kg, and 6960 ± 1710 mg/kg, respectively. These values confirmed a clear concentration gradient in the prepared sea mud feed matrices across the three glyphosate treatment groups.

#### 3.2.2. Tissue Residues and Compartmental Distribution

**Figure 3:**
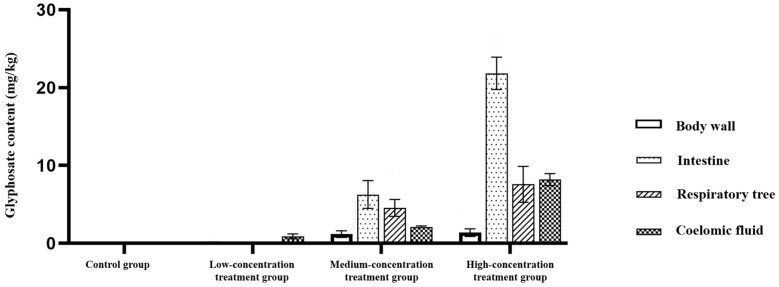
Tissue residues and compartmental distribution of glyphosate after 72 h exposure. Glyphosate concentrations in the body wall, intestine, respiratory tree, and coelomic fluid of *A. japonicus* after 72 h dietary/substrate-linked exposure. Data are presented as mean ± SD. Statistical evaluation was performed using two-way ANOVA with treatment group and tissue type as factors.

After 72 h of dietary/substrate-linked exposure, glyphosate residues were detected in all sampled tissues and body fluids of exposed animals, including the body wall, intestine, respiratory tree, and coelomic fluid (Figure 3). Residue levels varied across both treatment groups and tissue compartments. In the low-dose group, residue concentrations remained low and showed limited variation across tissue compartments. In contrast, the medium- and high-dose groups showed markedly elevated residue levels, with the respiratory tree and coelomic fluid tending to exhibit the highest concentrations. These patterns indicate that matrix-associated ingestion can produce measurable internal glyphosate residues within 72 h and that residue distribution is compartment-dependent.

#### 3.2.3. Statistical Evidence from Two-Way ANOVA

Two-way ANOVA showed significant effects of treatment group, tissue type, and their interaction on glyphosate residue distribution (Table 1). Thus, both treatment level and tissue compartment contributed to the observed residue distribution patterns, consistent with the trends shown in Figure 3.

### 3.3. Gut Microbiota Diversity, Taxonomic Composition, and Predicted Functional Profiles

#### 3.3.1. Taxonomic Composition Indicated a Strongly Dominance-Structured Microbiota

**Figure 4:**
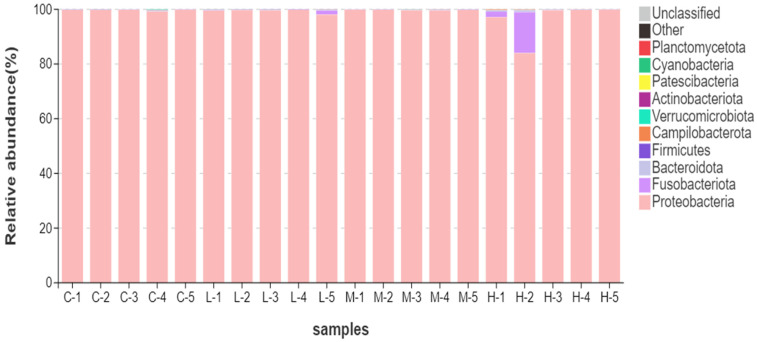
Sample-level relative abundance of dominant phyla in the intestinal microbiota. Stacked bar plots showing the relative abundance of the dominant phyla for each individual sample, labeled as C-1 to C-5, L-1 to L-5, M-1 to M-5, and H-1 to H-5, corresponding to the C, L, M, and H groups, respectively. Only the major phyla are shown, and the remaining low-abundance phyla are grouped as “Other” or “Unclassified”.

**Figure 5:**
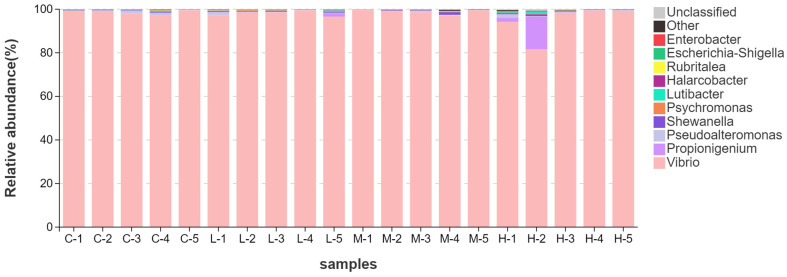
Sample-level relative abundance of dominant genera in the intestinal microbiota. Stacked bar plots showing the relative abundance of the dominant genera for each individual sample, labeled as C-1 to C-5, L-1 to L-5, M-1 to M-5, and H-1 to H-5, corresponding to the C, L, M, and H groups, respectively. Only the major genera are shown, and the remaining low-abundance genera are grouped as “Other” or “Unclassified”.

To improve the transparency of sample-level variation, relative abundance profiles were visualized for each individual sample at the phylum and genus levels (Figure 4 and Figure 5). At the phylum level, the intestinal microbiota was overwhelmingly dominated by Proteobacteria in most samples, whereas Fusobacteriota, Bacteroidota, and Firmicutes remained at much lower relative abundance. This dominance pattern was broadly consistent across the C, L, and M groups, whereas the H group showed greater within-group heterogeneity, mainly because one high-dose sample displayed a marked reduction in Proteobacteria accompanied by an increase in Fusobacteriota.

At the genus level, most samples were strongly dominated by *Vibrio*, while *Propionigenium*, *Pseudoalteromonas*, *Shewanella*, and *Psychromonas* occurred at substantially lower abundance. The dominance of *Vibrio* was largely maintained across the control, low-dose, and medium-dose groups, whereas one high-dose sample showed a noticeable reduction in *Vibrio* together with an increase in *Propionigenium*, resulting in greater heterogeneity within the H group. Taken together, these results indicate that the gut microbiota remained strongly dominance-structured across treatments, with limited broad-scale compositional turnover but detectable sample-level restructuring in part of the high-dose group.

#### 3.3.2. Alpha-Diversity Remained Broadly Comparable Across Treatments

**Figure 6:**
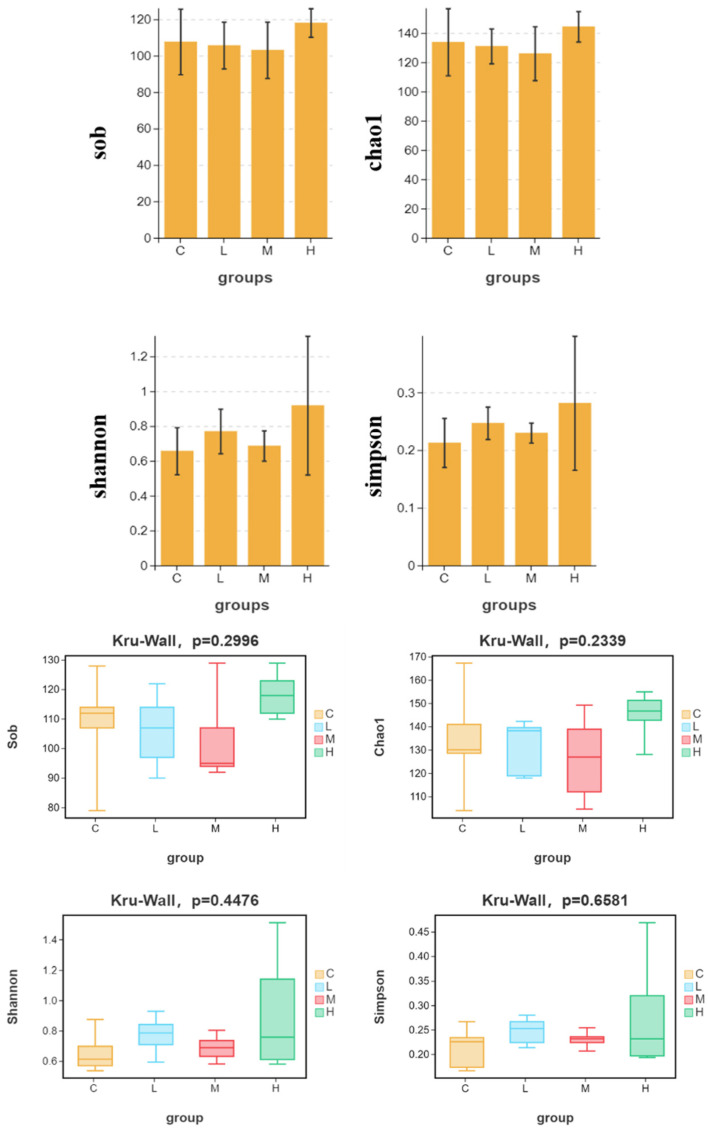
Alpha-diversity indices of the intestinal microbiota after 72 h dietary/substrate-linked exposure. Alpha-diversity indices, including Sobs, Chao1, Shannon, and Simpson were compared among the C, L, M, and H groups. Data are presented as boxplots with group-level distributions. Group differences were evaluated using the Kruskal–Wallis test.

Alpha-diversity analysis showed no statistically significant differences among the C, L, M, and H groups for Sobs, Chao1, Shannon, or Simpson indices (Kruskal–Wallis: Sobs, *p* = 0.2996; Chao1, *p* = 0.2339; Shannon, *p* = 0.4476; Simpson, *p* = 0.6681) (Figure 6). Although the H group tended to show slightly higher median values for some richness and diversity indices, within-group variation remained substantial, and the overall differences among treatments were limited within the 72 h exposure window.

#### 3.3.3. Beta-Diversity Showed Only Modest Treatment-Related Separation

**Figure 7:**
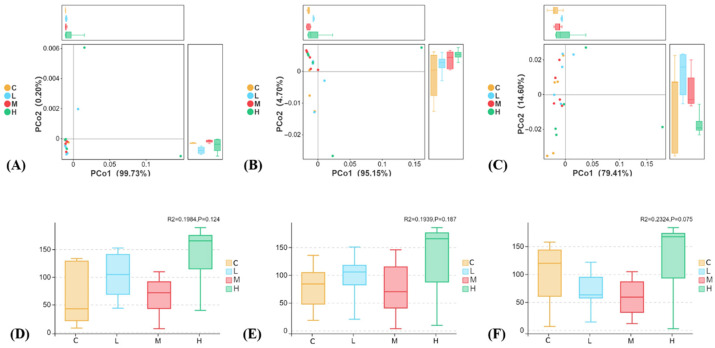
Beta-diversity patterns of the intestinal microbiota at different taxonomic resolutions. Principal coordinates analysis (PCoA) based on the Bray–Curtis distance was used to visualize between-group differences in a community structure. Panels (**A**–**C**) show the ordination patterns at different taxonomic resolutions, and panels (**D**–**F**) summarize the corresponding treatment-associated variation. Community differences among groups were evaluated using PERMANOVA.

Beta-diversity analysis revealed only modest treatment-associated separation among groups, and this pattern depended on the taxonomic resolution used for community summarization (Figure 7). At the coarser taxonomic resolution, treatment effects on community structure were weak (PERMANOVA R^2^ = 0.1984, *p* = 0.124; R^2^ = 0.1939, *p* = 0.167), whereas the finest-resolution analysis showed the largest treatment-associated shift, although it remained statistically non-significant under the current sample size (PERMANOVA R^2^ = 0.2324, *p* = 0.075). Overall, these results suggest that broad community turnover was limited, but some treatment-related restructuring may have occurred at finer compositional resolution.

#### 3.3.4. LEfSe Identified a Limited Set of Discriminatory Taxa

**Figure 8:**
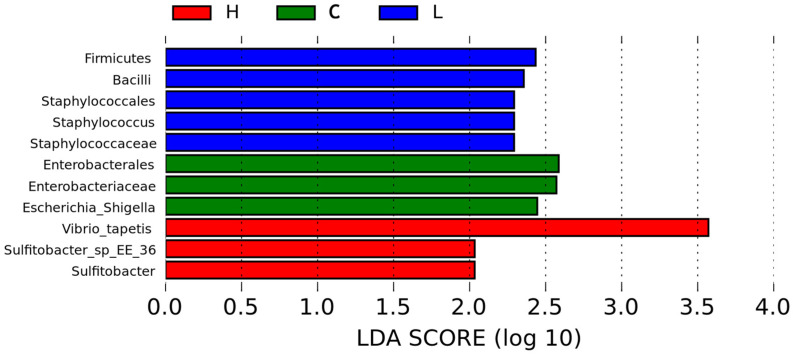
LEfSe analysis of differentially enriched taxa among treatment groups. Linear discriminant analysis (LDA) scores of taxa identified by LEfSe as differentially enriched among groups. Taxa with higher LDA scores contributed more strongly to group discrimination under the present analysis threshold.

**Figure 9:**
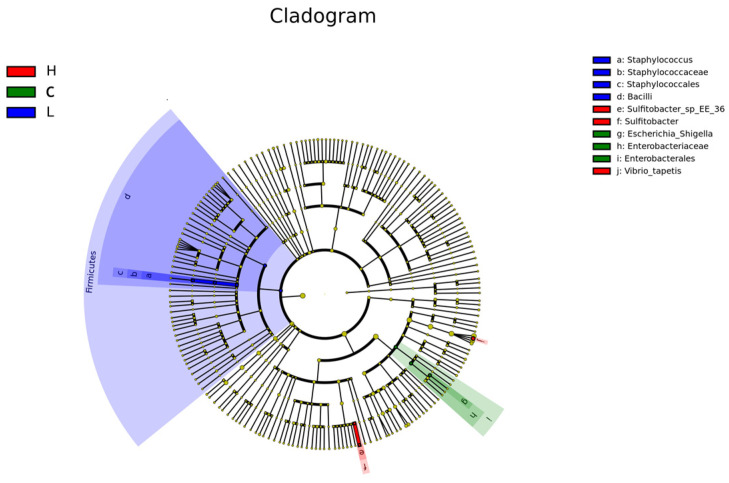
LEfSe cladogram showing taxonomic lineages differentially enriched among groups. Cladogram of differentially enriched taxa identified by LEfSe. Colored branches indicate taxa enriched in different treatment groups, whereas yellow nodes indicate taxa without significant differential enrichment.

LEfSe analysis identified a limited number of discriminatory taxa among groups (Figure 8 and Figure 9). Taxa enriched in the L group were mainly affiliated with *Firmicutes*, including *Bacilli*, *Staphylococcales*, *Staphylococcaceae*, and *Staphylococcus*. Taxa enriched in the C group were mainly associated with *Enterobacterales*, *Enterobacteriaceae*, and *Escherichia-Shigella*. In the H group, the main discriminatory taxa included *Sulfitobacter*, *Sulfitobacter_sp_EE_36*, and *Vibrio_tapetis*, with *Vibrio_tapetis* showing the highest LDA score among the detected biomarkers. No M-enriched biomarker taxa were detected under the present LEfSe threshold. Overall, these findings suggest that although the dominant community framework remained largely similar, a subset of lower-abundance lineages still showed treatment-associated enrichment patterns.

#### 3.3.5. PICRUSt2 Functional Prediction Indicated Broadly Similar Functional Profiles

**Figure 10:**
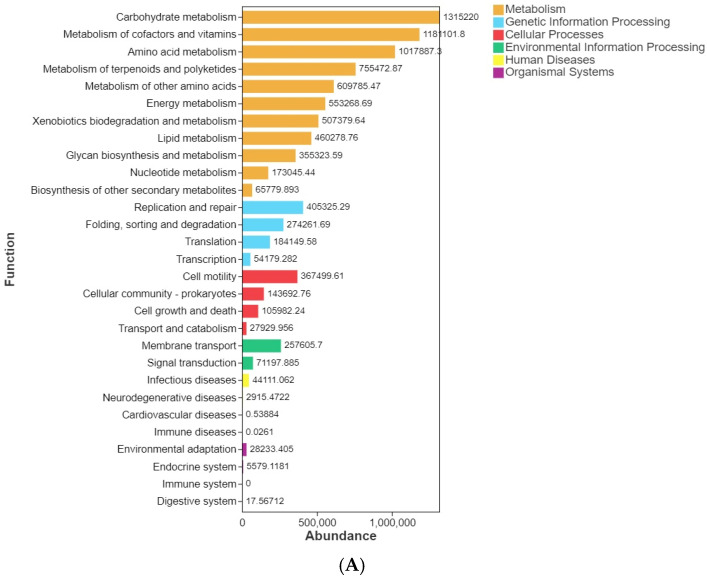

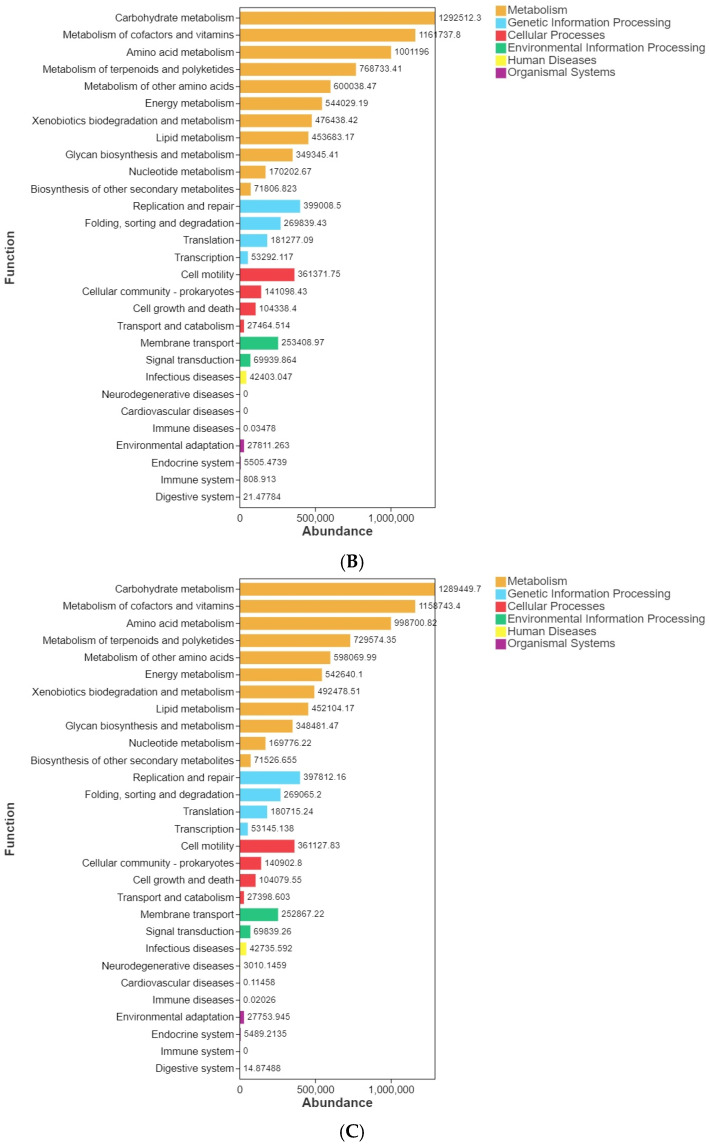

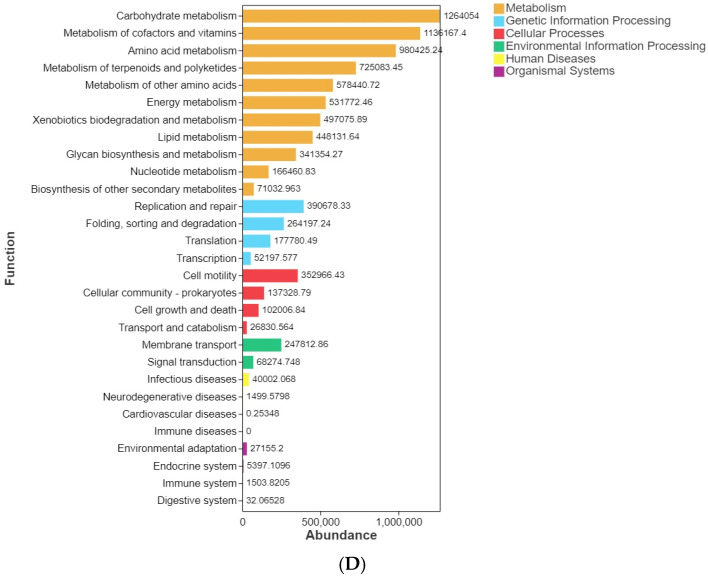
PICRUSt2-predicted functional profiles of the intestinal microbiota at KEGG level 2.Stacked bar plots showing the relative abundance of major KEGG level 2 functional categories across individual samples in the C, L, M, and H groups. Predicted functional profiles were generated using PICRUSt2 based on 16S rRNA gene sequencing data. (**A**) control (C), (**B**) low-dose (L), (**C**) medium-dose (M), and (**D**) high-dose (H) groups.

PICRUSt2-based functional prediction indicated broadly similar functional profiles across treatments (Figure 10). At KEGG level 2, the dominant predicted categories were consistently related to carbohydrate metabolism, metabolism of cofactors and vitamins, amino acid metabolism, metabolism of terpenoids and polyketides, metabolism of other amino acids, and energy metabolism, together with xenobiotics biodegradation and metabolism, lipid metabolism, replication and repair, and cell motility. The relative abundance structure of these major functional categories was highly similar among the C, L, M, and H groups, with only limited visual variation across samples. These findings suggest that the short-term dietary/substrate-linked exposure did not produce strong functional restructuring at the predicted community level within the present experimental window.

### 3.4. Digestive Enzymes: Largely Stable Responses with Selective Sensitivity in Amylase

**Figure 11:**
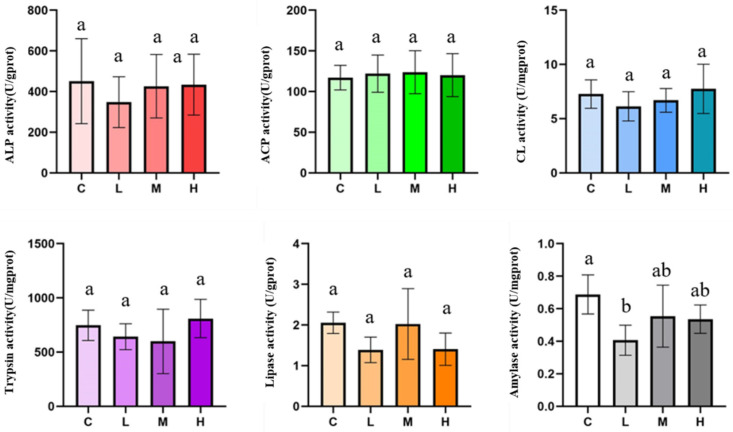
Digestive enzyme activities after 72 h exposure. Activities of digestive enzymes (ALP, ACP, CL, trypsin, LPS, and AMS) measured in intestinal extracts after 72 h of dietary/substrate-linked exposure. Data are presented as mean ± SD. Individuals were randomly selected from the endpoint sampling set described in Section 2.1. Statistical analysis was performed using one-way ANOVA, followed by appropriate post hoc comparisons where applicable. Significant differences among groups are indicated by different letters (a, b).

Digestive enzyme activities measured in intestinal extracts (*n* = 5 individuals per group) showed that ALP, ACP, cellulase (CL), trypsin, and lipase (LPS) did not differ significantly from the control (Figure 11). In contrast, amylase (AMS) showed a significant change in the low-dose (L) group relative to the control. Overall, these results indicate that short-term dietary/substrate-linked glyphosate exposure did not broadly alter digestive enzyme activity within the 72 h exposure window, whereas amylase appeared to be a comparatively more responsive endpoint under mild exposure conditions.

### 3.5. Immune and Antioxidant Biomarkers: SOD as the Primary Responsive Endpoint

**Figure 12:**
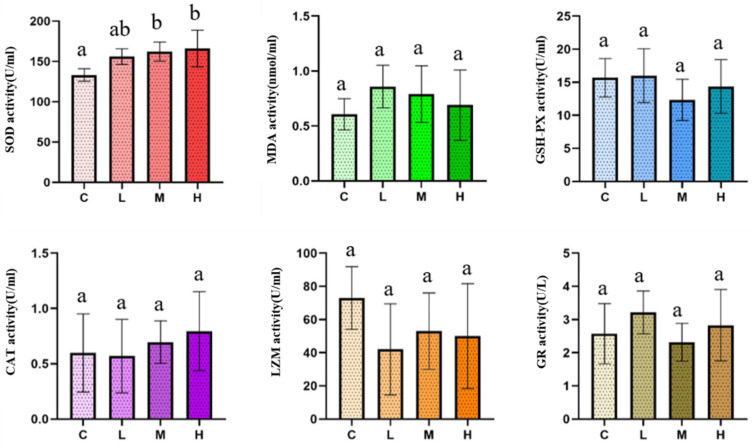
Immune and antioxidant biomarkers after 72 h dietary/substrate-linked exposure. Biomarkers measured in coelomic fluid (MDA, GSH-PX, CAT, LZM, GR, POD, and SOD) after 72 h dietary/substrate-linked exposure. Data are presented as mean ± SD. Individuals were randomly selected from the endpoint sampling set described in Section 2.1. Statistical analysis was performed using one-way ANOVA, followed by appropriate post hoc comparisons where applicable. Significant differences among groups are indicated by different letters (a, b).

Immune and oxidative stress-related biomarkers measured in coelomic fluid (*n* = 5 individuals per group) showed that MDA, GSH-PX, CAT, LZM, GR, and POD did not differ significantly among groups (Figure 12). In contrast, SOD showed dose-associated variation, with the medium-dose (M) group differing significantly from the control and the high-dose (H) group showing a stronger difference relative to the control. Collectively, these results indicate that most systemic immune and antioxidant biomarkers remained relatively stable during the 72 h exposure period, whereas SOD was the most responsive endpoint within this short-term exposure window.

## 4. Discussion

### 4.1. Sea Mud Matrix as a Likely Modifier of Dietborne Glyphosate Bioaccessibility and Apparent Short-Term Response Magnitude

A central finding of this study is that a sea mud feed matrix can modify dietborne glyphosate exposure by altering the relationship between external loading and apparent internal availability, thereby contributing to comparatively muted short-term physiological responses. In coastal systems, glyphosate and AMPA are repeatedly detected not only in water but also in particulate matter and sediments, indicating that benthic and deposit-feeding species may experience exposure through particle-associated fractions rather than freely dissolved forms alone [35,36,37]. Mechanistically, glyphosate is prone to strong interactions with mineral and clay surfaces through ligand exchange and complexation, and its adsorption behavior can be influenced by pH, ionic composition, and cation bridging, while phosphate may compete for adsorption sites and alter apparent uptake potential [29,38,39]. These processes provide a plausible explanation for why dietborne/substrate-linked exposure in a mud-rich matrix yielded measurable tissue residues but comparatively muted enzyme responses over 72 h. Matrix binding may constrain the freely bioaccessible fraction, slow uptake kinetics, and reduce peak internal exposure even when total external loads are high.

From the perspective of this study, this matrix-influenced exposure pathway should not be viewed as a trivial replication of waterborne paradigms. Instead, it represents a more husbandry-realistic scenario for deposit feeders that ingest sediment-like material, where the exposure route includes both solid-associated chemicals and gut-mediated processing. Therefore, comparatively weaker short-term responses may be interpreted as a route-dependent outcome related to exposure form and apparent bioaccessibility rather than as an indicator of low study value.

### 4.2. Tissue Residues Support Internal Exposure and Highlighting Route-Specific Toxicokinetics Interpretation

Despite the relatively limited physiological responses observed over 72 h, tissue measurements confirm internal exposure under the dietborne mud matrix design and indicate that glyphosate entered the organism and was distributed among tissues. Field monitoring in lagoon, estuarine, and river-to-sea contexts shows that glyphosate and AMPA can occur in water, suspended particulate matter, and sediments, reinforcing the plausibility of internal exposure in benthic organisms that contact and ingest particles [35,36]. At the same time, the sediment compartment can act as a reservoir for pesticide residues and degradation products, including AMPA, implying that internal exposure may occur under complex, particle-dominated conditions rather than under a purely dissolved-water exposure scenario [37]. Such environmental evidence strengthens the interpretation that dietborne residue signals can be ecologically meaningful even when short-term systemic disturbance appears limited.

Route-specific kinetics also provide a conceptual basis for the observed pattern of measurable residues but are modest biomarkers. In a mud matrix, glyphosate may be delivered to the gut partly in an adsorbed state, with desorption and release influenced by gut physicochemistry and competing ligands, which differs from direct gill or epithelial contact during waterborne exposure [38,39]. This distinction is particularly important for interpreting the present results: the toxicokinetic framing (external load → internal residue → comparatively muted short-term physiological responses) is consistent with matrix-influenced exposure and should be considered route-informative rather than redundant.

### 4.3. Relatively Limited Enzyme Responses Suggest Short-Term Physiological Adjustment Under a Likely Low-Bioaccessibility Exposure Regime

In this experiment, digestive and immune/oxidative enzyme activities collectively indicate that short-term systemic disturbance was relatively limited under sea mud–associated dietborne exposure. Enzyme biomarkers are widely used as sensitive readouts of sublethal stress in aquatic toxicology and aquaculture health assessment; however, the magnitude and detectability of enzyme responses often depend on exposure form, dose dynamics, and the degree to which the organism can maintain physiological homeostasis. Under a likely low-bioaccessibility scenario, organisms may maintain broadly stable enzyme activity over a short exposure period, with only selective endpoints responding (e.g., specific digestive enzymes under certain doses) rather than broad activation of oxidative or immune defenses.

Importantly, this relatively limited response pattern does not imply an absence of effect; rather, within the 72 h window of this short-term assay, it suggests that a mud-rich feeding context may attenuate acute systemic enzyme responses while internal residues still confirm uptake. Moreover, because glyphosate–mineral interactions and phosphate competition can modulate the amount of freely available glyphosate at the biological interface, the relatively limited enzyme responses observed here are consistent with known adsorption and competition behaviors, although direct quantification of the bioaccessible fraction will be needed to verify this mechanism more explicitly [38,39].

### 4.4. Dominance-Structured Microbiota Shifts May Provide Interface-Level Signals Even When Alpha Diversity Appears Uniformly Low

A notable feature of this dataset is the high dominance of a limited number of taxa (e.g., *Vibrio* and *Propionigenium*), which can yield low or weakly changing alpha-diversity metrics. However, low alpha diversity alone does not necessarily mean that microbiome responses are uninformative. Modern microbiome statistics emphasize that many gut datasets are sparse and compositional; community shifts can manifest as dominance restructuring and taxon-level replacement without large changes in richness or Shannon-type indices, especially when one taxon is already abundant [40,41]. Under such conditions, a “dominance-structured” interpretation—focusing on changes in dominant taxa, relative rank structure, and between-group compositional distances—may be more biologically informative than alpha-diversity comparisons alone.

This framing is particularly relevant for deposit-feeding invertebrates whose gut communities may be strongly shaped by ingested particles and sediment-derived microbes. Evidence from sea cucumber studies under other stressors suggests that gut microbiota can undergo restructuring and potential functional shifts that align with host stress physiology, even when some summary metrics appear relatively insensitive [42,43,44]. In addition, glyphosate is known to influence microbial pathways, both directly in microbes and indirectly through host–microbe interactions, and microbiome-level sensitivity has been documented across animal systems, supporting the plausibility that microbial community structure can shift under exposure even when organism-level endpoints show only modest changes [45]. Therefore, the microbiota results in this study may be interpreted as an “interface layer” signal, which is a potentially earlier or more sensitive indicator of exposure influence than short-term systemic enzyme disruption.

### 4.5. Beta-Diversity Patterns and PERMANOVA Support Community-Level Differences, with Dispersion Checks Strengthening Interpretation

The beta-diversity ordination outcomes (PCoA based on Bray–Curtis dissimilarity) and PERMANOVA results suggest community-level differences in overall composition, complementing the taxon-level dominance patterns described above. Distance-based community analyses are standard in microbial ecology and are appropriate for detecting multivariate shifts that may not be captured by single-metric summaries alone [46]. At the same time, PERMANOVA can be influenced by group dispersion (heterogeneity of within-group distances), so reporting and discussing a dispersion check (PERMDISP) helps clarify whether the observed differences are more consistent with centroid separation than with changes in within-group spread alone [47,48]. Taken together, the combination of Bray–Curtis dissimilarity, PCoA ordination, PERMANOVA testing, and dispersion assessment provides a more complete framework for interpreting microbiome variation under low-diversity, dominance-structured conditions.

From a biological perspective, a scenario in which beta-diversity differences are detectable while alpha-diversity changes remain subtle is plausible and increasingly recognized, especially in compositional datasets where dominance shifts, replacement, or restructuring within a few taxa can move samples in multivariate space without dramatically altering within-sample diversity [40,41]. Thus, the beta-diversity and PERMANOVA results strengthen the interpretation that dietborne exposure in a sea mud matrix may influence gut community structure even when traditional enzyme biomarkers show only a limited short-term response.

### 4.6. PICRUSt2 Functional Predictions Are Hypothesis-Generating and Broadly Consistent with a Dominance-Structured Interpretation

PICRUSt2 provides a practical approach for inferring functional profiles from 16S data, and it is most appropriately treated here as a hypothesis-generating layer that complements taxonomy and beta-diversity rather than replacing direct metagenomic evidence [49,50]. In a dominance-structured community, predicted functions are likely to be influenced strongly by the dominant lineages, which can still be informative if interpreted cautiously. Apparent functional differences may reflect shifts in the metabolic potential of the most abundant taxa rather than broad community-wide remodeling. This interpretation is also consistent with the present results, in which dietary exposure was associated with subtle but structured microbiome variation under a likely low-bioaccessibility scenario.

To keep the interpretation appropriate and conservative, it is helpful to emphasize that predicted functional shifts indicate candidate pathways for follow-up validation (e.g., targeted qPCR of key genes, metagenomics, or metabolite readouts), and that the predictions remain sensitive to reference databases, phylogenetic placement, and community composition [49,50]. Reporting PICRUSt2 alongside transparent microbiome processing and analysis workflows is also consistent with current reproducible microbiome analytical practices used in the field [51,52]. Overall, the PICRUSt2 layer adds a useful exploratory dimension to the microbiome findings while still requiring cautious interpretation of inference strength.

### 4.7. Implications for Aquaculture Health Assessment Under Benthic Exposure Routes Relevant to Husbandry Conditions

For sea cucumber aquaculture, one practical interpretation is that sea mud-dominated feeding contexts may limit overt short-term systemic responses to glyphosate exposure, while measurable internal residues and microbiota restructuring indicate that exposure remains biologically engaged. In practical terms, short-term survival and external appearance may remain normal while gut ecological balance shifts, a pattern consistent with a subclinical response in which the host maintains gross health status while the gut interface begins to reorganize. Given that field monitoring repeatedly documents glyphosate and AMPA in particle-rich compartments, particularly in systems receiving terrestrial inputs, route-aware risk assessment should consider sediment and matrix binding as an important modifier of exposure and apparent effect size [35,36,37]. This also implies that husbandry-related factors affecting sediment ingestion intensity, organic load, and matrix composition influence modulate realized risk, highlighting a management-relevant consideration beyond the simple presence or absence of environmental glyphosate.

### 4.8. Limitations and Future Directions

Several limitations should be acknowledged to support the interpretation of the present findings and to clarify priorities for future work. First, the present work focuses on a short (72 h) exposure window; the relatively limited enzyme responses observed here may reflect short-term physiological adjustment that could shift under longer exposure durations, repeated dosing, or additional stressors. Second, the dominance-structured and low-diversity character of the microbiota warrants careful analytical interpretation; inclusion of extraction/PCR blanks and explicit reporting of PERMDISP alongside PERMANOVA would further strengthen interpretability in future work [47,48]. Third, 16S-based functional inference is not a substitute for direct functional measurements; PICRUSt2 results should be treated as prioritized hypotheses for downstream validation [49,50]. Finally, expanding endpoints that connect gut changes to host condition (e.g., histology, barrier markers, and targeted metabolites) would strengthen causal interpretation, as suggested by sea cucumber studies in which gut microbiota restructuring co-occurs with physiological disruption under other stressors [42,43,44]. Collectively, these steps help clarify whether the present pattern of limited short-term systemic responses together with gut-interface restructuring persists under longer or repeated exposure scenarios and would strengthen route-specific mechanistic interpretation.

## 5. Conclusions

This study demonstrates that a sea mud feed matrix can shape dietborne glyphosate exposure in the deposit-feeding sea cucumber (*Apostichopus japonicus*), resulting in measurable internal residues but largely buffered short-term physiological responses over 72 h. No mortality or obvious external lesions were observed, while tissue residues confirmed uptake and compartmental distribution under the matrix-linked route. Most digestive and immune/antioxidant biomarkers remained stable, with amylase and superoxide dismutase emerging as the most responsive endpoints within this short exposure window. Gut microbiota analyses revealed a dominance-structured community in which alpha-diversity metrics were insensitive to treatment, whereas compositional and community-structure signals were more evident at finer taxonomic resolution; predicted functional potentials were broadly conserved across groups. Collectively, these results highlight that sediment-associated glyphosate exposure may manifest as subtle, gut-interface–centered changes prior to overt health impairment, underscoring the value of integrating residue verification with microbiome-aware metrics for aquaculture health assessment. Future work should quantify the bioaccessible glyphosate fraction in the mud–gut context and evaluate longer or repeated exposures to determine whether buffered responses persist or progress to chronic effects.

## Figures and Tables

**Table 1:** Two-way ANOVA for glyphosate residues across treatment groups and tissue compartments.

Source	df	F	*p*
Treatment group	3	260.0	<0.0001
Tissue type	3	88.74	<0.0001
Treatment × Tissue	9	54.57	<0.0001

Table note: Statistical evaluation was performed using two-way ANOVA with treatment group and tissue type as factors.

## Data Availability

All data supporting the findings of this study are contained within the article.
